# Resveratrol–Curcumin Hybrid Selectively Induces Chromosomal Abnormalities and Apoptosis in Colon Adenocarcinoma Cells

**DOI:** 10.3390/antiox14111367

**Published:** 2025-11-17

**Authors:** Mariane Minussi Baptistella, Aléxia Polo Siqueira, Dâmaris Lizia Santos Magalhães, Bruno Zavan, Carolina Sales de Oliveira, Matheus de Freitas Silva, Ellen Tardelli Falleiros Lima, Claúdio Viegas, Bruno Martins Dala-Paula, Ester Siqueira Caixeta, Marisa Ionta, Pollyanna Francielli de Oliveira

**Affiliations:** 1 Instituto de Ciências da Natureza, Universidade Federal de Alfenas, Alfenas 37130-001, MG, Brazil; 2 Instituto de Ciências Biomédicas, Universidade Federal de Alfenas, Alfenas 37130-001, MG, Brazil; 3 Instituto de Química, Universidade Federal de Alfenas, Alfenas 37130-001, MG, Brazil; 4 Faculdade de Nutrição, Universidade Federal de Alfenas, Alfenas 37130-001, MG, Brazil

**Keywords:** molecular hybridization, HCT-8, cell cycle arrest, *AURKB*, *CDKN1A*

## Abstract

Colorectal cancer (CRC) therapy frequently relies on chemotherapeutic agents with high cytotoxicity, low selectivity, and suboptimal efficacy. Thus, the search for alternative therapeutic strategies for CRC continues. In the present work, the antitumor potential of a hybrid compound, which contains fragments derived from resveratrol and curcumin, was evaluated. These natural compounds are known by their antioxidant, chemopreventive, and chemotherapeutic properties. Different methodologic approaches were used to investigate cytotoxic, genotoxic, antiproliferative, and antioxidant effects of a hybrid compound, named PQM-162, on HCT-8 colorectal cancer cells. The results showed that PQM-162 displays radical scavenging capacity as demonstrated by DPPH assay. Furthermore, this substance reduced cell viability and inhibited cell cycle progression at G2/M in HCT-8 cells. Antiproliferative activity of PQM-162 was associated with its ability to modulate the expression of critical regulators of G2/M transition and mitosis progression such as *PLK1*, *AURKB*, and *CDKN1A*. Taken together, our data indicate that PQM-162 is a promising antitumor agent due to its disruption of the redox balance in cancer cells and its modulation of the expression of regulators of the cell cycle and mitotic apparatus.

## 1. Introduction

According to Global Cancer Statistics 2022, colorectal cancer (CRC) was the third-most diagnosed cancer (1,926,118 new cases—9.6%) and the second cause of cancer-related death worldwide (903,859 new deaths—9.3%) [1]. Reflecting both the growth and aging of the population, the number of deaths due to CRC is expected to increase by 70–75% by 2035. In addition, inaccessibility of early detection methods and specialized care contribute to CRC development and progression [2]. The most diagnosed CRCs are sporadic and have no direct association with genetic predisposition or family history (20–30%) [3]. The accumulation of genetic and epigenetic alterations is crucial for CRC development, which is influenced by environmental factors such as smoking, a sedentary lifestyle, high intake of red and processed meat, and low intake of fiber and vitamins. Male sex and increasing age (50+) are associated with an increasing incidence of CRC [4,5].

CRC is often diagnosed at an advanced stage [4,5], and clinical management typically involves systemic therapy. Since the antimetabolite 5-fluorouracil (5-FU; C4H3FN2O2) was introduced into clinical practice in 1957, it has become the primary treatment strategy [5,6]. Commonly, 5-FU is used in combination with other therapeutic agents, such as irinotecan, oxaliplatin, leucovorin, bevacizumab, cetuximab, and panitumumab, to improve the therapeutic response. However, drugs used in systemic therapy are not completely effective and promote many side effects. Thus, it is crucial to amplify the therapeutic arsenal for CRC treatment [6,7]. Although promising outcomes of immunotherapy have been reported, the response rates are still insufficient [8]. Therefore, the development of new drugs and therapeutic approaches has been prioritized to overcome the drug’s resistance [6]. In this sense, the antitumor potential of natural compounds has been investigated, and medicinal chemists have worked to find innovative prototypes with more effective antitumor activity.

**Figure 1:**
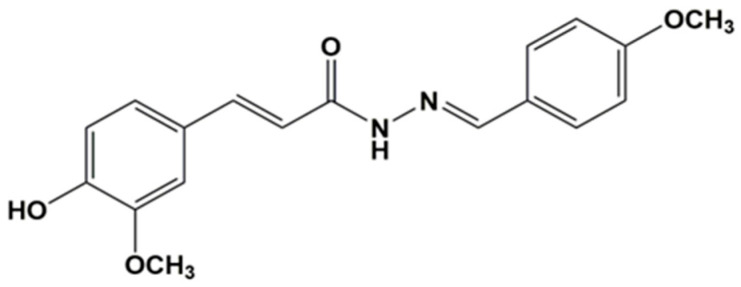
Chemical structure of the resveratrol–curcumin hybrid PQM-162 (*E*)-3-(4-hydroxy-3-methoxyphenyl)-*N*’-((*E*)-4-methoxybenzylidene) acrylohydrazide.

Resveratrol and curcumin are recognized for their capacity to modulate various oncogenic signaling pathways [9]. It has been demonstrated that resveratrol can inhibit all stages of carcinogenesis (initiation, promotion, and progression) [10], and curcumin demonstrates antiproliferative, anti-inflammatory, antimetastatic, antiangiogenic, and proapoptotic activity [10,11]. Despite the promising antitumor effects of these natural compounds, they cannot be used in clinical practice due to their poor bioavailability [10]. To overcome this limitation, de Freitas et al. [12] synthesized a series of resveratrol and curcumin hybrids using the molecular hybridization technique to obtain a substance with antitumor potential and druggable features. The authors demonstrated that (*E*)-3-(4-hydroxy-3-methoxyphenyl)-*N*’-((*E*)-4-methoxybenzylidene) acrylohydrazide (named PQM-162, Figure 1) inhibited, with higher potency, the proliferation of estrogen-positive breast adenocarcinoma (MCF7) compared with natural prototypes of resveratrol and curcumin by inducing mitosis arrest and apoptosis [12]. Herein, the main objective was to evaluate the antitumor potential of PQM-162 against colorectal adenocarcinoma cells (HCT-8). We investigated the antiproliferative, cytotoxic, and genotoxic effects and the mechanism of action and predictive ADMET properties of PQM-162.

## 2. Materials and Methods

### 2.1. Synthesis

The molecule (*E*)-3-(4-hydroxy-3-methoxyphenyl)-*N*’-((*E*)-4-methoxybenzylidene) acrylohydrazide (PQM-162; MM: 327.26 g/mol) was synthesized and chemically characterized according to the methodology previously published by de Freitas Silva et al. [12] by the PeQuiM (Laboratory of Research in Medicinal Chemistry of Federal University of Alfenas). The synthesis was based on the preparation of the key hydrazide intermediates, which were coupled to a series of substituted benzaldehydes, leading to the desired curcumin–resveratrol hybrid compound (PQM-162). This substance exhibits a methoxy group in substitution for a hydroxy group at the resveratrol-derived moiety; this is used to evaluate their pharmacophoric contribution to the antiproliferative activity.

### 2.2. Determination of Antioxidant Potential

The antioxidant potential was determined by spectrophotometric assays based on the decolorization of the ABTS^•+^ cation and DPPH radicals, resulting from the scavenging activity of antioxidant compounds in the sample. Results were expressed as µmol Trolox equivalents (µmol TEs) per mmol of sample. The data are presented as mean ± SD (standard deviation) from three independent experiments.

The DPPH free radical scavenging assay was conducted by adding 100 μL of appropriately diluted sample to a test tube containing 650 μL of a 0.1 mmol/L DPPH (2,2-diphenyl-1-picrylhydrazyl) [13] solution prepared in methanol. To construct the calibration curve, various concentrations of the standard compound Trolox (6-hydroxy-2,5,7,8-tetramethylchroman-2-carboxylic acid) ranging from 1.0 to 14.7 nmol/mL were used. The reaction mixtures were kept protected from light for 30 min at 25 °C, after which the absorbance was determined at 517 nm.

For the ABTS^•+^ assay, the radical cation solution was generated by combining 5000 μL of a 7 mmol/L solution of ABTS^•+^ [2,2′-azino-bis (3-ethylbenzothiazoline-6-sulfonic acid)] with a 2.45 mM potassium persulfate solution [13,14]. This mixture was incubated in the dark for 12 h at 25 °C. The resulting ABTS^•+^ stock solution was then diluted with ultrapure water until reaching an absorbance of 0.700 ± 0.005 at 734 nm, generating the working solution. To perform the assay, 250 μL of the properly diluted sample was added to 750 μL of the ABTS^•+^ working solution in a test tube, and absorbance was measured at 734 nm using a spectrophotometer. Trolox standard curves were established using concentrations between 1 and 17 nmol/mL.

### 2.3. Cell Lines and Culture Conditions

The colorectal adenocarcinoma cell line (HCT-8, BCRJ-0106) and an immortalized human keratinocyte cell line (HaCaT, BCRJ-0341), which were purchased from Rio de Janeiro cell Bank, were used in the present study. The cell lines were periodically tested for mycoplasma contamination. The HCT-8 cell line was chosen based on its established use as a model of aggressive colorectal adenocarcinoma and its high proliferative capacity. Both adherent cell cultures were maintained as monolayers in culture flasks (25 cm^2^) containing 5 mL of DMEM (Dulbecco’s Modified Eagle Medium, Sigma-Aldrich, Burlington, MA, USA), supplemented with 10% fetal bovine serum (FBS) and 1% penicillin and streptomycin, in a humidified atmosphere of 5% CO_2_ at 37 °C. Subcultivation using trypsin/EDTA 0.2% was performed every other day, with 80–90% cell confluence. Cells from 4th to 12th passage were used.

### 2.4. Treatment Schedule

5-Fluorouracil (5-FU–MM: 130.08 g/mol) was purchased from Sigma-Aldrich and used as positive control (PC). The stock solutions of 5-FU (77 mM) and PQM-162 (46 mM) were prepared in dimethylsulfoxide (DMSO, Sigma-Aldrich) solvent and stored at −20 °C. The substances were diluted in culture medium immediately before use to obtain final concentrations. The amount of the vehicle in culture medium (*v*/*v*) did not exceed 1% for functional assays. The cells were seeded into plates containing 24 or 6 wells, depending on the experimental approach. After attachment (24 h), the cells were treated for 24 or 48 h. The experiments were conducted in triplicate, and appropriate controls were included. The data are presented as mean ± SD from at least three independent experiments.

### 2.5. Sulforhodamine B (SRB) Viability Assay

HCT-8 cells were seeded into 96-well tissue culture plates, at a density of 5 × 10^3^ cells, in 100 µL DMEM/F12 medium. After attachment, the cells were treated with PQM-162 at concentrations ranging from 1 to 20 μM for 48 h. Solvent control (DMSO 0.1%) was used as negative control. The cells were fixed with 10% trichloroacetic acid (TCA) and incubated for 60 min at 4 °C to allow protein precipitation and fixation. Fixed cells were stained with SRB solution (0.4% in 1% acetic acid) and incubated for 30 min at room temperature. After repeated washes in 1% TCA, the samples were dried, and SRB stably bound to cellular proteins was solubilized in 10 mM of Tris-base (30 min). The absorbance was measured at 540 nm, with a reference wavelength of 690 nm, using a microplate reader. Cell viability for each treated well was calculated as a percentage relative to the vehicle control. The IC_50_ values were calculated using GraphPad Prism^®^ 8.0 software (GraphPad Software, Inc., San Diego, CA, USA). Experiments were conducted in quadruplicate, and the results are presented as mean ± SD from at least three independent experiments.

### 2.6. Colony Formation Assay

Cells at a density of 1 × 10^5^ cells were seeded in 6-well tissue culture plates and treated with PQM-162 or 5-FU. PQM-162 was used at 5; 10 and 20 μM for HCT-8 and at 20 and 40 μM for HaCaT cells. The 5-FU was used in a concentration range of 0.2–0.8 μM for both cell lines. After 48 h, the cultures were trypsinized, and 500 cells were seeded from each sample in another plate to recover in a drug-free medium for the next seven days. After fixation, Giemsa-stained colonies (>50 cells) were visualized and counted at 20× magnification in stereomicroscopy. The data are presented as mean ± SD from three independent experiments.

### 2.7. Cytokinesis-Block Micronucleus Assay (CBMN-Cyt)

The quantification of chromosomal abnormalities was carried out according to OECD 487 guideline [15] and Fenech’s [16] established protocol. A doubling time of 15 and 24 h was considered for HCT-8 and HaCaT, respectively. A total of 5 × 10^5^ cells were seeded. After 30 h for HCT-8 and 48 h for HaCaT, cultures were exposed to the treatments. HCT-8 cells were treated with PQM-162 at 20 and 40 μM concentrations, and HaCaT cells were treated with PQM-162 at 30 and 60 μM concentrations. 5-FU [0.8 μM] was used as a DNA damage-inducing agent (PC). Untreated cultures were used as negative control (NC) and DMSO 1% as a solvent control (SC). After 48 h, the treatments were removed, and the cultures were incubated with cytochalasin-B (Cyto-B, 3 μg/mL). After 22 and 36 h, for HCT-8 and HaCaT, respectively, the cells were fixed and transferred to a microscope slide and stained with 3% Giemsa for 3 min. The analyses were performed by light microscopy, using a 100× immersion objective.

The mean frequency of micronuclei (MN) was scored and expressed as the number of events per 1000 binucleated cells for each replicate, resulting in 3000 per group. The nuclear division index (NDI) was calculated to determine proliferation and cytostatic effects, according to Eastmond and Tucker [17]: NDI—[(1 × M1 + 2 × M2 + 3 × M3 + 4 × M4)/N] where cells with 1, 2, 3, and 4 nuclei are M1 to M4, respectively, and N represents the count of viable cells—was evaluated by the score of 500 cells per replicate (total of 1500 per group) with preserved cytoplasm. The data are presented as mean ± SD from three independent experiments.

### 2.8. Cell Cycle Analysis

HCT-8 cells were seeded into 6-well tissue culture plates at a density of 1 × 10^5^. Treatment was performed for 24 h with PQM-162 at 10 and 20 μM. The cells were collected by enzymatic digestion (trypsin/EDTA, Sigma-Aldrich) and centrifuged at 1000 rpm for 5 min. Cell pellets were obtained, and samples were fixed with 75% ethanol in PBS (phosphate-buffered saline) for 30 min. Afterwards, cells were homogenized in dye solution [PBS containing 90 mg mL^−1^ propidium iodide (PI) and 1.5 mgmL^−1^ RNAase]. The analysis was performed using a flow cytometer (Guava^®^ easyCyte™ 8HT, Hayward, CA, USA). The data are presented as mean ± SD from three independent experiments.

### 2.9. Apoptosis Detection by Annexin-V FITC/PI

Annexin-V FITC/PI kit (Invitrogen, Waltham, MA, USA) was used according to the manufacturer’s instructions. Briefly, cells were seeded into 24-well tissue culture plates at a density of 1 × 10^5^ cells per well. Cultures were treated with PQM-162 (at 20 and 40 μM) and cisplatin (at 60 μM) for 24 h. Thereafter, cells were collected by enzymatic digestion (Trypsin-EDTA solution, Sigma-Aldrich), centrifuged at 1000 rpm for 5 min, washed in ice-cold PBS, and homogenized in a solution containing annexin conjugated to FITC (fluorescein isothiocyanate) and propidium iodide. The samples were incubated for 20 min and protected from light at room temperature, and the analysis was performed using a flow cytometer (Guava^®^ easyCyte™ 8HT, Hayward, CA, USA). The data are presented as mean ± SD from three independent experiments.

### 2.10. Transcript Level Evaluation

Total RNA was isolated using the RNeasy^®^ Micro Kit (Qiagen, Mississauga, ON, Canada), following the protocol provided by the manufacturer, and subsequently eluted in 30 μL of RNase-free water. The RNA yield was quantified via spectrophotometry using the NanoDrop^®^ ND 1000 device (Thermo Fisher Scientific, Wilmington, DE, USA). For the removal of residual genomic DNA, 1 µg of total RNA was treated with DNase I (1 U/µg; Invitrogen). Following this step, cDNA synthesis was performed using random primers and the High-Capacity cDNA Reverse Transcription Kit (Applied Biosystems, Waltham, Massachusetts, USA), as per the manufacturer’s recommendations (see Supplementary Materials, Table S1). The reverse transcription reaction was carried out under the following thermal conditions: 25 °C for 10 min, 37 °C for 120 min, and a final incubation at 85 °C for 5 min to deactivate the enzyme.

Quantification of gene expression for *CDKN1A*, *CDK1*, *PLK1*, *CCNB1*, *AURKA*, and *AURKB* was performed by quantitative real-time PCR using an ABI 7500 system and Power SYBR™ Green PCR Master Mix (Applied Biosystems). Primer sequences are provided in Table S1 (Supplementary Materials). Each reaction was carried out in a 25 μL final volume. The amplification program included an initial denaturation at 95 °C for 10 min (1 cycle), followed by 40 cycles of 95 °C for 10 s and 60 °C for 1 min for annealing and extension.

To normalize the expression levels of the target genes, *ACTB* (β-actin) was used as the endogenous control. Relative gene expression was determined using the ΔΔCt method, incorporating reaction efficiency and calibration with a control sample [18]. Amplification efficiency for each gene was calculated using the LinRegPCR v. 2015.4 software based on individual amplification curves [19]. The data are presented as mean ± SEM with four independent experiments.

### 2.11. Immunofluorescence

HCT-8 cells were seeded at a density of 1 × 10^5^ cells per 35 mm Petri dish on coverslips and treated with PQM-162 for 48 h. After treatment, cells were fixed in 3.7% formaldehyde for 30 min, and plasma membrane was permeabilized with Triton X-100 (0.5% in PBS) for 15 min. Samples were incubated with the primary antibody [anti-α-tubulin (1:1000, Cell Signaling Technology—#3873)] overnight at 4° C. Next day, the samples were incubated with secondary antibodies for 2 h at room temperature [anti-mouse IgG-FITC antibody (1:100 Sigma-Aldrich)]. For actin visualization, samples were incubated with phalloidin-TRICT conjugated (Sigma-Aldrich) for 2 h. Nuclei were counterstained with DAPI in a Fluoroshield mounting medium (Sigma-Aldrich). Images were acquired using a laser scanning confocal microscope (AX, Nikon, Tokyo, Japan) and NIS Elements software. The data are presented as mean ± SD from three independent experiments.

### 2.12. Alkaline Comet Assay

The alkaline comet assay was carried out following the foundational protocol by Singh et al. [20], with some modifications. In summary, HCT-8 cells were plated in culture flasks and exposed to PQM-162 at concentrations of 10 or 20 μM for 48 h. Following incubation, cells were harvested using enzymatic dissociation, and their viability was assessed via the trypan blue exclusion method. Only cell suspensions with viability exceeding 70% were processed. A total of 2 × 10^5^ viable cells were gently mixed with 100 μL of 0.5% low-melting-point agarose (Sigma-Aldrich) maintained at 37 °C, and this mixture was evenly spread onto slides previously coated with 1.5% normal-melting-point agarose (Sigma-Aldrich).

Slides were then immersed in a cold lysis buffer (4 °C) composed of 1% Triton X-100, 10% DMSO, 2.5 mM of NaCl, 100 mM of Na_2_EDTA, and 100 mM of Tris (pH 10) and kept under these conditions for 24 h. Electrophoresis was performed under alkaline conditions (25 V, 300 mA) for 25 min. After this step, the slides were neutralized using 0.4 M Tris–HCl (pH 7.5) for 15 min and fixed with ethanol. For visualization, nucleoids were stained with SYBR^®^ Green (Invitrogen, Thermo Fisher Scientific) diluted 1:100 in 1× PBS. Fluorescence microscopy at 20× magnification was used for image acquisition.

Quantitative analysis of DNA damage was conducted using the OpenComet plugin in the ImageJ v1.3.1 software (ImageJ, Wayne Rasband, NIH, USA), enabling measurement of tail and head intensities of each comet. Ultraviolet (UV) radiation was employed as a positive control. Comets were imaged, and comets meeting any of the following exclusion criteria were omitted from analysis: overlapping comets, comet “ghosts” with no identifiable head, comets with fragmented heads, and comets with saturated signal. For each condition, 50 nucleoids were randomly selected and evaluated per slide. The extent of DNA damage was expressed as tail moment (mean ± SD), calculated as tail moment = (tail length) × (% DNA in tail), based on three independent replicates.

### 2.13. Prediction Study

The prediction study was performed using QikProp v. 3.5 (Schrödinger, LLC, NY, 2018). Physicochemical and pharmacokinetic properties for PQM-162 were determined, and these values were compared with those of resveratrol and curcumin parental compounds.

### 2.14. Statistical Analysis

Statistical significance was determined using one-way analysis of variance (ANOVA), followed by Dunnett post-test. The values are presented as the mean ± standard deviation (SD) or mean ± standard error of mean (SEM) of at least three independent experiments. Furthermore, a comparative analysis of the effects between the cell lines in the CBMN-cyt assay was carried out using a nested fixed effects analysis of variance model, fitted in a completely randomized design. While negative control was used as a criterion for analysis, *p* < 0.05 was considered statistically significant.

## 3. Results

### 3.1. Antioxidant Potential

The antioxidant activity of PQM-162 was evaluated using ABTS^•+^ and DPPH radical scavenging assays. The compound exhibited a moderate capacity to neutralize the ABTS^•+^ radical, with a Trolox equivalent value of 26.36 ± 3.34 nmol TE/µmol molecule. In contrast, a substantially higher antioxidant capacity was observed in the DPPH assay, yielding 382.82 ± 7.29 nmol TE/µmol molecule (Table 1).

### 3.2. Cytotoxic and Antiproliferative Investigation

**Figure 2:**
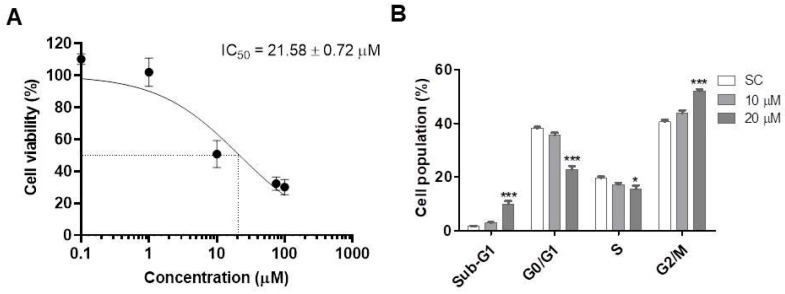
**PQM-162 reduces viability and induces cell cycle arrest at G2/M in HCT-8 cells.** (**A**) Cell cultures were treated with PQM-162 at different concentrations for 48 h, and cell viability was assessed by sulforhodamine B. Results are presented as the mean ± standard deviation (SD) from three independent experiments. IC_50_ value was determined from a nonlinear regression curve. (**B**) Cell cycle analysis was performed after treatment with PQM-162 at concentrations around IC_50_ and IC_50_-/2. PQM-162: (*E*)-3-(4-hydroxy-3-methoxyphenyl)-*N*’-((*E*)-4-methoxybenzylidene) acrylohydrazide. SC: solvent control (DMSO—dimethyl sulfoxide 1%). * *p* < 0.05; *** *p* < 0.001.

The PQM-162 reduced the viability rate in a concentration-dependent manner after 48 h of treatment (Figure 2A). The IC_50_ value for PQM-162 on HCT-8 was around 20 µM. Cell cycle analyses revealed that PQM-162 at IC_50_ concentration inhibits cell cycle progression at G2/M as demonstrated by a significant increase in the G2/M population, with reduction in the G1 and S populations (Figure 2B).

**Figure 3:**
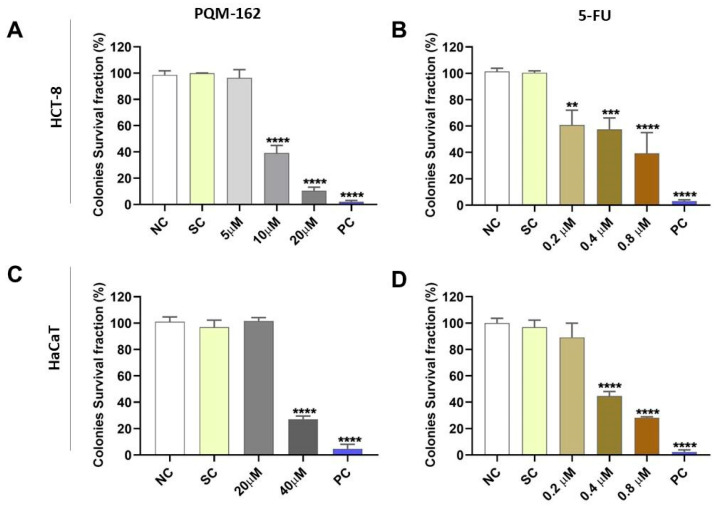
**PQM-162 inhibits the long-term cell survival of HCT-8 tumor cells.** HCT-8 (**A**,**B**) and HaCaT (**C**,**D**) cells were treated with PQM-162 or reference drug 5-FU, respectivelly for 48 h and recovered in fresh medium for 7 days. Results are presented as the mean ± standard deviation (SD) from three independent experiments. Significant differences from negative control group (NC) were determined according to ANOVA analysis followed by Tukey’s multiple comparisons test. ** *p* < 0.01; *** *p* < 0.001, **** *p* < 0.0001. HCT-8: colorectal adenocarcinoma cell line, HaCaT: normal human keratinocytes cell line, PQM-162: (*E*)-3-(4-hydroxy-3-methoxyphenyl)-*N*’-((*E*)-4-methoxybenzylidene) acrylohydrazide, 5-FU: 5-fluorouracil, SC: solvent control (DMSO—dimethyl sulfoxide 1%), PC: positive control (DMSO—dimethyl sulfoxide 25%).

Based on the viability data, we evaluated the long-term antiproliferative activity of PQM-162 on HCT-8 cells through a clonogenic assay (Figure 3). In this experimental approach, we included non-tumor cells (HaCaTs) and a reference drug, 5-FU, that is a powerful cytotoxic drug used in chemotherapy for CRC. The results showed that the survival fraction was significantly reduced in HCT-8 samples treated with PQM-162 at 10 μM or 20 μM (Figure 3A). By contrast, in HaCaT cell cultures, only the samples treated with PQM-162 at 40 μM exhibited a reduced survival fraction (Figure 3C). As expected, 5-FU drastically inhibited the clonogenic capacity in both HCT-8 and HaCaT cell lines at very low concentrations (0.2–0.8 µM range for HCT-8 and 0.4–0.8 µM for HaCaT) (Figure 3).

**Figure 4:**
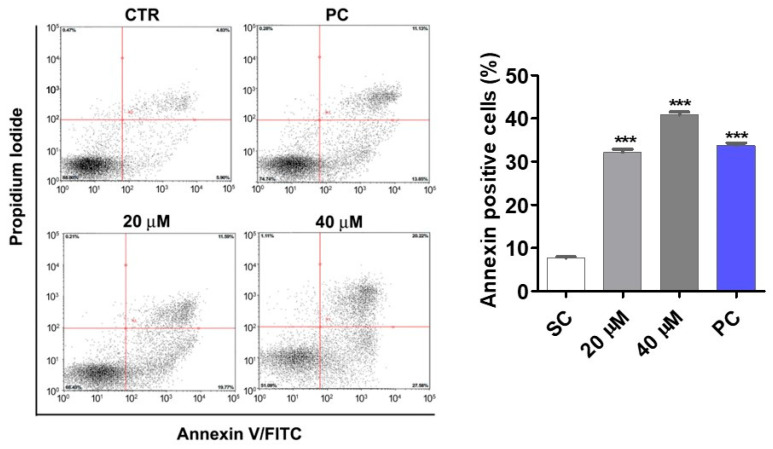
**PQM-162 induces apoptosis in HCT-8 tumor cells.** HCT-8 cells treated with PQM-162 for 48 h at 20 or 40 µM. Results are presented as the mean ± standard error of the mean (SEM) from three independent experiments. Significant differences from the negative control group (SC) were determined according to ANOVA analysis followed by Dunnett’s multiple comparisons test. *** *p* < 0.001. PQM-162: (*E*)-3-(4-hydroxy-3-methoxyphenyl)-*N*’-((*E*)-4-methoxybenzylidene) acrylohydrazide. SC: solvent control (DMSO—dimethyl sulfoxide 0.1%), PC: positive control (cisplatin at 60 µM).

Since PQM-162 at 20 µM induced a significant increase in Sub-G1 population in HCT-8 cultures, we performed an annexin V assay to evaluate the proapoptotic activity of this substance on HCT-8 cells. Increased frequencies of positive cells for annexin V were observed in groups treated with PQM-162 at 20 and 40 µM (Figure 4).

### 3.3. Genotoxic Potential Investigation

**Figure 5:**
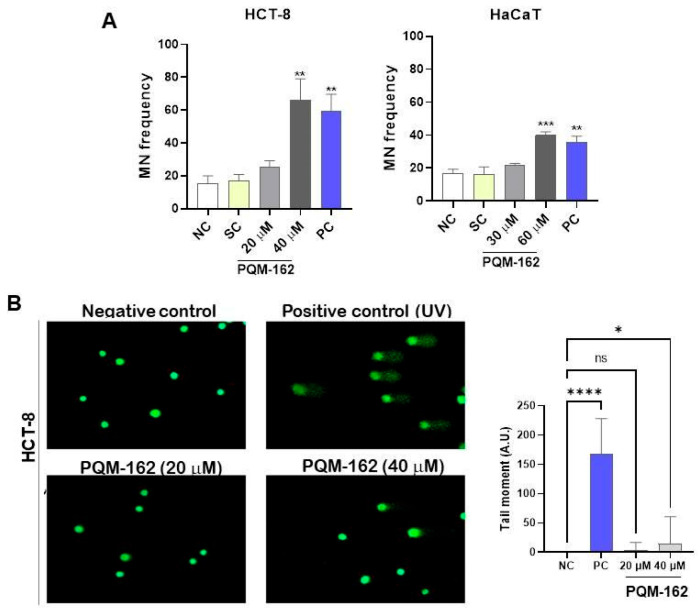
**PQM-162 at high concentrations has a genotoxic effect on HCT-8 cells.** (**A**) Micronucleus (MN) frequency was determined after 48 h of treatment with PQM-162 in HCT-8 and HaCaT cell lines. A total of 1000 binucleated cells were counted per slide, resulting in a total of 3000 events per treatment. Results are presented as the mean ± standard deviation (SD from three independent experiments. Significant differences from negative control groups were determined according to ANOVA analysis, followed by Dunnett’s multiple comparisons test. ** *p* < 0.01 and *** *p* < 0.001. PQM-162: (*E*)-3-(4-hydroxy-3-methoxyphenyl)-*N*’-((*E*)-4-methoxybenzylidene) acrylohydrazide. NC: negative control, SC: solvent control (DMSO—dimethyl sulfoxide 0.5%), PC: positive control (5-FU—5-fluorouracil-0.8 µM). (**B**) Representative images and quantitative data from the alkaline comet assay performed at 48 h of treatment. Fifty nucleoids were quantified per slice, resulting in a total of 150 nucleoids per treatment. Values are presented as the mean ± standard error of the mean (SEM). * *p* < 0.05 and **** *p* < 0.0001 according to ANOVA analysis followed by Dunnett’s multiple comparisons test comparing treated samples with NC group. PC: positive control (ultraviolet light exposure for 5 min).

The nuclear division index in the cytokinesis-block micronucleus assay (CBMN-cyt) was approximately 1.5, indicating that PQM-162 did not alter the nuclear division index under the experimental conditions used (Figure S1). No significant differences were observed in micronuclei frequencies between the control and PQM-162-treated (20 µM) groups; however, when PQM-162 was used at high concentrations (40 µM for HCT-8 and 60 µM for HaCaT), increased micronuclei frequencies were observed in treated samples compared to controls (Figure 5A). Additionally, the ability of PQM-162 to induce primary DNA damage in HCT-8 at 20 and 40 µM was investigated through the comet assay. Consistent with previous data, PQM-162 induced DNA damage in HCT-8 cells only in samples treated with 40 µM (Figure 4B). The positive control groups treated with (5-FU) and UV radiation revealed, respectively, a significant increase in the frequency of micronuclei and DNA damage when compared to the negative control group, validating the experimental design for this assay.

### 3.4. Molecular Investigation and Cytoskeleton Analysis

**Figure 6:**
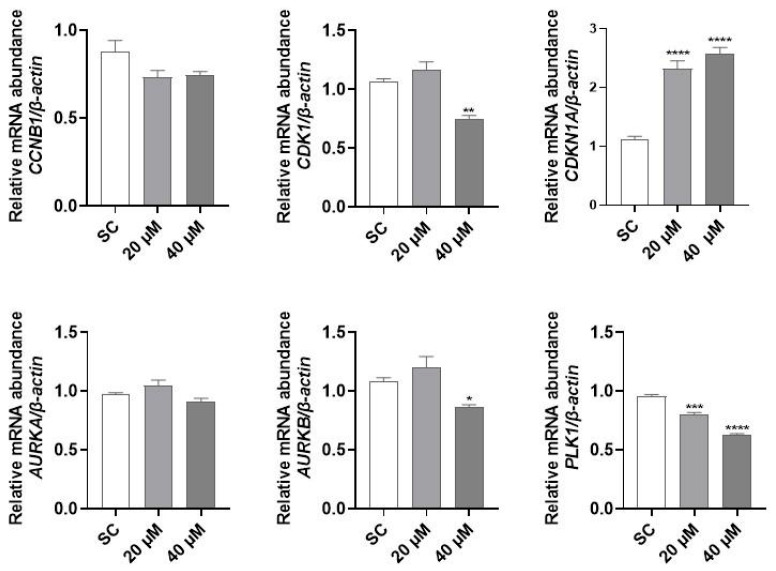
**PQM-162 modulates gene expression of regulators of cell cycle in HCT-8 cells.** Cells were treated with PQM-162 at 20 or 40 µM for 24 h, and mRNA abundance was determined by qPCR. Results are presented as the mean ± standard deviation (SD) from three independent experiments. Significant differences from control groups (SC) were determined according to ANOVA followed by Dunnett’s post-test. * *p* < 0.05, ** *p* < 0.01, *** *p* < 0.001, **** *p* < 0.0001. PQM-162: (*E*)-3-(4-hydroxy-3-methoxyphenyl)-*N*’-((*E*)-4-methoxybenzylidene) acrylohydrazide. SC: solvent control (DMSO—dimethyl sulfoxide 0.1%).

Since PQM-162 altered the dynamic of cell cycle progression and promoted cell cycle arrest at G2/M, gene expression profiles of regulators of G2/M transition and mitosis onset were assessed. PQM-162 at 20 µM induced a prominent upregulation of *CDKN1A* (p21) and a downregulation of *PLK1*. The same effects were observed in samples treated with PQM-162 at 40 µM; however, in this concentration, mRNA abundance for *AURKB* (Aurora B) was also reduced compared to the SC control group (Figure 6).

**Figure 7:**
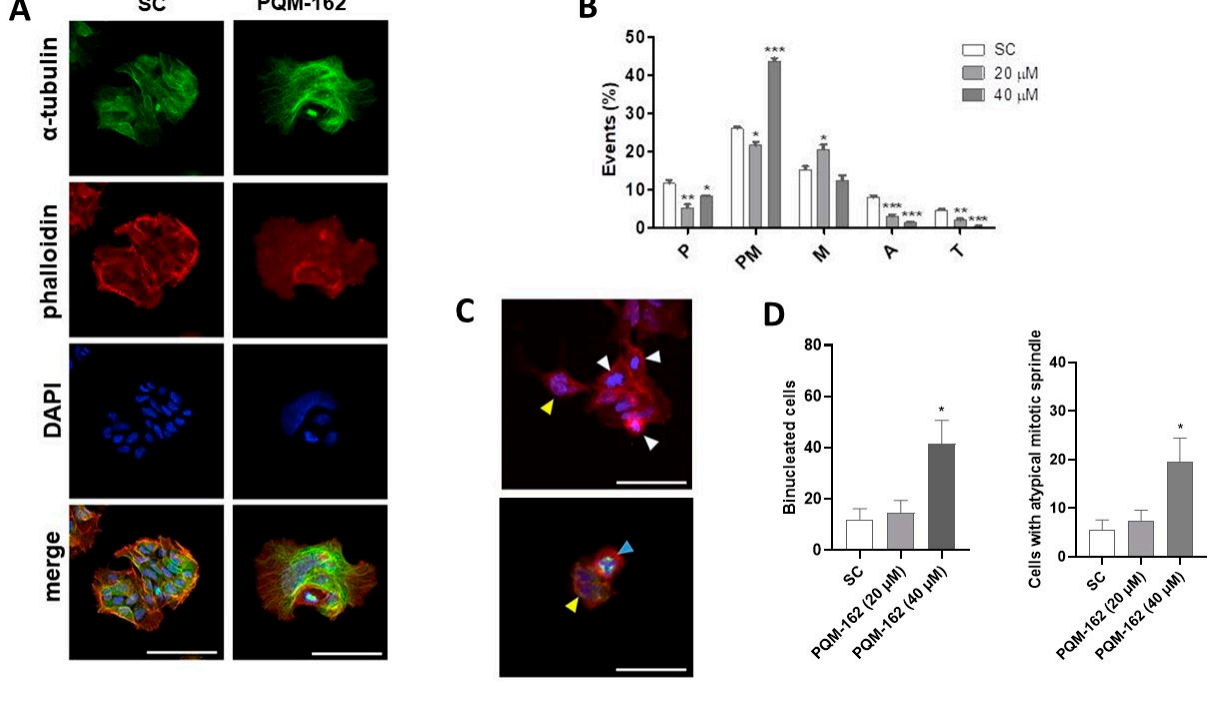
**PQM-162 disturbs the distribution pattern of microtubules and microfilaments in HCT-8 cells.** Cells were treated with PQM-162 for 48 h at 20 or 40 µM, and fluorescent cytological preparations labeled for microtubule and microfilaments were observed by confocal microscopy. Microtubules were evidenced by anti-α-tubulin-Alexa-488 conjugation, and microfilaments were identified via labeling with phalloidin-TRITC conjugation. Nuclei were stained with DAPI. (**A**) Representative images obtained by confocal microscopy showing microfilament and microtubule distribution patterns in control and PQM-162-treated (20 µM) cells, scale bar: 50 μm. (**B**) Mitotic index showing the cell population distributed across different mitotic subphases. P: prophase; PM, prometaphase; M, metaphase; A, anaphase; and T, telophase. Results are presented as the mean ± standard deviation (SD) from three independent experiments. Significant differences compared to the control were determined according to ANOVA followed by Dunnett’s post-test. * *p* < 0.05, ** *p* < 0.01, *** *p* < 0.001. (**C**) Representative images of HCT-8 cells treated with PQM-162 at 40 µM. Cells in prometaphase (white arrowhead), binucleated cells (yellow arrowhead), and a cell with a tripolar spindle (blue arrowhead), scale bar: 50 μm. (**D**) Quantitative data concerning binucleated and abnormal mitotic spindle. PQM-162: (*E*)-3-(4-hydroxy-3-methoxyphenyl)-*N*’-((*E*)-4-methoxybenzylidene) acrylohydrazide. SC: solvent control (DMSO—dimethyl sulfoxide 0.1%).

The mitotic index revealed that PQM-162 disrupts mitosis dynamics. The frequencies of cells in metaphase (20 µM) and prometaphase (40 µM) were significantly higher in the treated samples compared with the control groups. Non-treated cells exhibited microtubule networks throughout the cytoplasm (Figure 7A). By contrast, in cells treated with PQM-162 at 40 µM, the microtubules were concentrated in a specific region of the cytoplasm. Apparently, PQM-162 affected the dynamic instability of microtubules, leading to their stabilization. Likewise, the actin microfilament distribution pattern was altered by PQM 162 treatment. In control cultures, the actin cytoskeleton was clearly observed in both the cytoplasm and cortical regions. In treated samples, actin filaments were barely detectable, suggesting that PQM 162 promoted microfilament disruption (Figure 7A). Additionally, an increased frequency of enlarged cells, binucleated cells, and cells displaying abnormal mitotic spindles was observed in samples treated with PQM-162 at 40 µM (Figure 7C,D).

### 3.5. Prediction Study

According to the prediction study, PQM-162 displays characteristics that are important for oral drug candidates. Through QikProp, 17 physicochemical and pharmacokinetic descriptors/properties were determined for PQM-162, which were compared with the parental compounds. The parameters determined might provide insights into important aspects such as likeness, blood–brain permeability, solubility, oral absorptivity, etc. A summary of these parameters is shown in Table 1. PQM-162 was estimated to be better than curcumin and resveratrol in solubility (AcceptHB; 4.75), permeability (QPPCaco; 724.02), and human oral absorption (0 to 100% scale) (100%). The predicted values of PQM-162 for all properties were within ideal ranges, and it did not violate Lipinski’s rule of five and Jorgensen’s rule of three. Among these descriptors, #star provides the number of parameter values that fall outside the range for 95% of the test set. PQM-162 had parameter values within the range of 95% (Table 2).

## 4. Discussion

Five-fluorouracil (5-FU) is an important chemotherapeutic drug for colorectal cancer (CRC) treatment. However, it is highly cytotoxic, causing several side effects, and its therapeutic efficacy is limited [21,22]. In the present study, we evaluated the effects of a curcumin–resveratrol hybrid (PQM-162) on colon cancer cells (HCT-8). The design of hybrid molecules that harbor different pharmacophore fragments within the same scaffold represents an interesting approach to obtaining new multi-target drug candidates [23,24]. Previously, we demonstrated that PQM-162 was able to inhibit the proliferation of estrogen-positive breast cancer cells (MCF-7) by inducing cell cycle arrest at mitosis onset with minimal effects on normal cells [12]. We have recently reported the chemopreventive effect of PQM-162 on colon preneoplastic lesions by targeting the Wnt/β-catenin signaling pathway, as well as anti-inflammatory and antioxidant pathways [25]; however, its ability to act as a chemotherapeutic agent has not been demonstrated. In the present study, we explored the antitumor potential of PQM-162 and demonstrated that it exhibits antioxidant, antiproliferative, and proapoptotic effects on CRC cells.

PQM-162 was more effective in radical scavenging against DPPH compared to ABTS^•+^, suggesting a possible difference in the compound’s reactivity depending on the type of radical species generated. Our findings reinforce the conclusion that PQM-162 might trigger antioxidant response as demonstrated in a previous study performed with an *in vivo* model. Such antioxidant capacity may contribute to modulating oxidative stress within the tumor microenvironment, thereby affecting redox-sensitive signaling pathways involved in cell cycle progression and survival, although the effectiveness of this strategy has not yet been well established [26,27,28].

In a previous study, PQM-162 was tested on fibroblasts derived from healthy skin under the same experimental conditions used in the present work, and IC_50_ found was approximately 158 µM; i.e., its cytotoxic profile on normal cells is much lower compared with CRC HCT-8 cells. Interestingly, PQM-162 at 10 µM reduced the colony frequency by approximately 50% in HCT-8 cultures, whereas no effect was observed in keratinocytes (HaCaT) treated with this substance up to 20 µM. Additionally, when PQM-162 was tested on primary fibroblast cells, its cytotoxic effect was minimal, with IC_50_ around 150 µM [12]. These findings suggest that PQM-162 displays selectivity toward tumor cells, in contrast to the reference drug 5-FU, which significantly reduced the clonogenic capacity of both tumor and non-tumor cells. Although our study does not aim to position PQM-162 as a substitute for 5-FU, these findings support further investigation.

Cell cycle analysis revealed that PQM-162 inhibited cell cycle progression at G2/M in HCT-8 cultures. In addition, we observed a significant increase in the frequency of cells with fragmented DNA (sub-G1) in response to 20 µM of PQM-162. Consistent with these data, we observed increased frequency of apoptotic cells in HCT-8-treated cell cultures. Importantly, we did not observe significant increase in necrosis events after treatment, even when a remarkably high concentration (60 µM) was used. Cell cycle arrest at G2/M and apoptosis induction in colon cancer cells have been reported for curcumin and resveratrol natural compounds [29,30,31], but it is well documented that these compounds have unfavorable pharmacokinetic proprieties [32,33]. The introduction of the hydrazone spacer between two aromatic rings in PQM-162 could improve its solubility coefficient and bioavailability compared to curcumin and resveratrol [34,35]. Thus, we performed a prediction study to explore absorption, distribution, and permeability (ADME) properties for PQM-162. The results showed that the curcumin–resveratrol hybrid compound had an improved pharmacokinetic profile compared to the parental compounds. The same can be observed in relation to antioxidant activity: the resveratrol, a well-characterized phenolic antioxidant, exhibits potent radical scavenging activity consistent with approximate values observed for PQM-162 [36,37]. Meanwhile, curcumin demonstrated comprehensive antioxidant activity across multiple assays, including DPPH and ABTS, albeit still significantly less than recorded for PQM-162 [38,39].

The evaluation of cell cycle regulatory genes showed that PQM-162 promotes upregulation of *CDKN1A* (encoding p21, a pan CDK inhibitor protein) and downregulation of *PLK1* (encoding serine/threonine PLK1 kinase) and *AURKB* (encoding serine/threonine Aurora B kinase) in a concentration-dependent manner. The p21 plays key roles in several cellular events, including cell cycle regulation, apoptosis, gene transcription, and DNA repair. Regarding cell cycle dynamic, nuclear p21 inhibits cyclin-dependent kinases (CDKs)/cyclins complexes, especially cyclin B/CDK1 and CDK2/cyclin E, promoting cell cycle arrest in response to different types of cellular stress such as DNA damage. At transcriptional levels, p21 can be regulated by either p53-dependent or p53-independent pathways [40,41]. We demonstrated that PQM-162 induces DNA damage in HCT-8 cells at concentrations up to IC50; however, the molecular mechanism underlying the prominent p21 transactivation in HCT-8 cells in response to PQM-162 at IC_50_ concentration will be further elucidated. *PLK1* is a regulator of cell cycle progression at multiple stages, such as mitotic entry, spindle formation, chromosome segregation, and cytokinesis [42]. Importantly, various tumor tissues display overexpression of *PLK1*, which has been correlated with poor prognosis [43,44]. Recent studies have shown that *PLK1* may be a therapeutic target for CRC [45,46]. It has been reported that pharmacological inhibition of *PLK1* in colon carcinoma cells (HTC-116) induces p21 upregulation, leading to cell cycle arrest and, in turn, apoptosis or senescence depending on cellular context [47,48]. Interestingly, the increased expression of p21 was observed in a 5-FU-resistant CRC cell line (HCT-8/5-FU) in response to treatment with ethanolic extract of *Scutellaria barbata* D. [49]. Additionally, Yuan et al. [50] reported that the activation of the p53/p21/CDK1/cyclin B1 axis induced by suppression of ubiquitin-specific protease 39 (USP39) leads to inhibition of proliferation and colony-formation activities of HCT-116 cells. The authors suggest that USP39 promotes colon cancer proliferation in vitro and in vivo tumorigenesis by negatively regulating p21. Our data showed that PQM-162 effectively induced upregulation of p21 in HCT-8, which seems to be critical for cell cycle arrest and apoptosis induction.

Notably, PQM-162 at 40 µM displays mutagenic effects on HCT-8 cells, as evidenced by increasing MN frequency in PQM-162-treated cultures. Consistent with previous data, normal cells were less susceptible to the genotoxic effects of PQM-162 compared to tumor cells. Notably, PQM-162 at 40 µM concomitantly induced downregulation of three genes critical for G2/M transition and mitosis progression (*PLK1*, *CDK1*, and *AURKB*). Our findings suggest that the high cytotoxicity of PQM-162 on HCT-8 cells at concentrations above IC_50_ may be associated with the modulation of mitotic kinases critical for centrosome maturation, bipolar spindle formation, and accurate chromosomal segregation.

## 5. Conclusions

In the present study we demonstrated that PQM-162 promoted upregulation of p21 with concomitant downregulation of *PLK1* leading to cell cycle arrest and apoptosis. These findings support further investigation to evaluate direct molecular targets of PQM-162 in cancer colon cells and drug combination studies to validate its antitumor potential.

## Figures and Tables

**Table 1:** Antioxidant potential of PQM-162 by ABTS^•+^ and DPPH radical scavenging assays.

Analytical Parameters (nmol TE/µmol Molecule)	PQM-162
ABTS^•+^	26.36 ± 3.34
DPPH	382.82 ± 7.29

ABTS^•+^: 2,2′-azino-bis (3-ethylbenzothiazoline-6-sulfonic acid); DPPH- 2,2-diphenyl-1-picrylhydrazyl; TE: Trolox equivalent (µmol/g). PQM-162: (*E*)-3-(4-hydroxy-3-methoxyphenyl)-*N*’-((*E*)-4-methoxybenzylidene) acrylohydrazide. Data represent mean values ± standard deviation (SD) in triplicate (*n* = 3).

**Table 2:** QikProp descriptor/property values calculated for resveratrol–curcumin hybrid (PQM-162) and parental compounds.

Descriptor/Property	Compounds			Range/Recommended Values
	PQM-162	Resveratrol	Curcumin	
Reactive FG	1	0	2	0–2
CNS	−2	−2	−2	−2 to +2
mol_MW	326.35	228.24	368.38	130–725
DonorHB	2	3	2	0–6
AccptHB	4.75	2.25	7	2.0–20.0
QPlogPo/w	3.23	13.64	19.38	−2 to 6.5
QPlogS	−4.33	−2.75	−4.51	−6.5 to 0.5
QPlogHERG	−6.03	−5.29	−6.39	below −5
QPPCaco	724.02	279.84	234.42	<25: poor; >500: great
QPlogBB	−1.21	−1.28	−2.03	−3–1.2
QPlogKhsa	0.12	−0.17	−0.01	−1.5–1.5
Human oral absorption	3	3	3	1,2,3: low, med, high
% Hum Oral Absorption	100	8.36	86.53	>80%: high <25%: poor
PSA	87.71	67.49	110.3	7.0–200.0
Rule of 5	0	0	0	max 4
Rule of 3	0	0	0	max 3
#Stars	0	0	0	0–5

Reactive FG: number of reactive functional groups. The presence of these groups can lead to false positives in high-throughput screening assays and to decomposition, reactivity, or toxicity problems in vivo. CNS: central nervous system activity on a −2 (inactive) to +2 (active) scale. mol. MW: molecular weight. DonorHB: estimated number of hydrogen bonds that would be donated by the solute to water molecules in an aqueous solution. Values are averages obtained over several configurations, so they can be non-integers. AccptHB: estimated number of hydrogen bonds that would be accepted by the solute from water molecules in an aqueous solution. Values are averages obtained over several configurations, so they can be non-integers. QPlogPo/w: octanol/water partition coefficient. QPlogS: aqueous solubility, log S. S in mol dm–3 is the concentration of the solute in a saturated solution that is in equilibrium with the crystalline solid. QPlogHERG: IC50 value for blockage of HERG K+ channels. QPPCaco: apparent Caco-2 cell permeability in nm/sec. Caco-2 cells are a model for the gut–blood barrier. QPlogBB: brain/blood partition coefficient. QPlogKhsa: binding to human serum albumin. Human oral absorption: qualitative human oral absorption: 1, 2, or 3 for low, medium, or high. % Hum Oral Absorption: human oral absorption on 0 to 100% scale. PSA: van der Waals surface area of polar nitrogen and oxygen atoms. Rule of 5: number of violations of Lipinski’s rule of five (mol_MW < 500, QPlogPo/w < 5, donorHB ≤ 5, accptHB ≤ 10). Rule of 3: number of violations of Jorgensen’s rule of three (QPlogS > −5.7, QPPCaco > 22 nm/s, # primary metabolites < 7). #Stars: number of property or descriptor values that fall outside the 95% range of similar values for known drugs. PQM-162: (*E*)-3-(4-hydroxy-3-methoxyphenyl)-*N*’-((*E*)-4-methoxybenzylidene) acrylohydrazide.

## Data Availability

The original contributions presented in this study are included in the article/Supplementary Material. Further inquiries can be directed to the corresponding author.

## Supplementary Materials

The following supporting information can be downloaded at: https://www.mdpi.com/article/10.3390/antiox14111367/s1, Figure S1: Nuclear Division Index determined after 48 hours of treatment with PQM-162. Five hundred binucleated cells were counted per slide resulting in a total of 1500 binucleated cells in HaCaT (**A**) and HCT-8 (**B**) cytological preparations. Values are presented as the mean ± standard deviation (SD) of three independent experiments. ANOVA analysis followed by Tukey’s multiple comparisons test. HCT-8—colorectal adenocarcinoma cell line, HaCaT – normal human keratinocytes cell line, NC—negative control, SC—solvent control (DMSO—dimethyl sulfoxide 1%), PC—positive control (5-FU—5-fluorouracil-0.8 µM), NDI—Nuclear Division Index, PQM-162 (*E*)-3-(4-hydroxy-3-methoxyphenyl)-*N*′-((*E*)-4-methoxybenzylidene) acrylohydrazide. Table S1: Information of specific primers used for amplification in real-time PCR.
