# Supplementary material for: Resveratrol–Curcumin Hybrid Selectively Induces Chromosomal Abnormalities and Apoptosis in Colon Adenocarcinoma Cells
Source: Antioxidants (Basel). 2025 Nov 17;14(11):1367. doi: 10.3390/antiox14111367 (PMC12649679; doi:10.3390/antiox14111367)
Supplement: Supplementary file 1 [file antioxidants-14-01367-s001.zip › antioxidants-3899466-supplementary.pdf]

Supplementary Material

Ta**ble S1.** Information of specific primers used for amplification in real-time PCR.

Gene	Sequence	Reference
CDKN1A	F 5'- CCATAGCCTCTACTGCCACCATC-3'<br>R 5'- GTCCAGCGACCTTCCTCATCCA-3'	NM_001291549.1
CDK1	F 5'- ATGAGGTAGTAACACTCTGG-3'<br>R 5'- CCTATACTCCAAATGTCAACTG-3'	NM_001786.4
CCNB1	F 5'- GTACCCTCCAGAAATTGGTGA -3'<br>R 5'- GACTACATTCTTAGCCAGGTG -3'	NM_031966.2
PLK1	F 5'- CCTGCACCGAAACCGAGTTAT -3'<br>R 5'- CCGTCATATTCGACTTTGGTTGC -3'	NM_005030.5
AURKA	F:5'- TCTTCACAGGAGGCAAATCCA -3'<br>R:5'- AATAAGTTACACACTCACTCAGGTACTA -3'	NM_003600.3
AURKB	F:5'- AAAGAGCCTGTCACCCCATC -3'<br>R:5'- CGCCCAATCTCAAAGTCATC -3'	XM_017025309.1
ACTB	F 5'- AGAGCTACGAGCTGCCTGAC-3'<br>R 5'- AGCACTGTGTTGGCGTACAG-3'	NM_001101.3

F = forward primer; R = reverse primer.

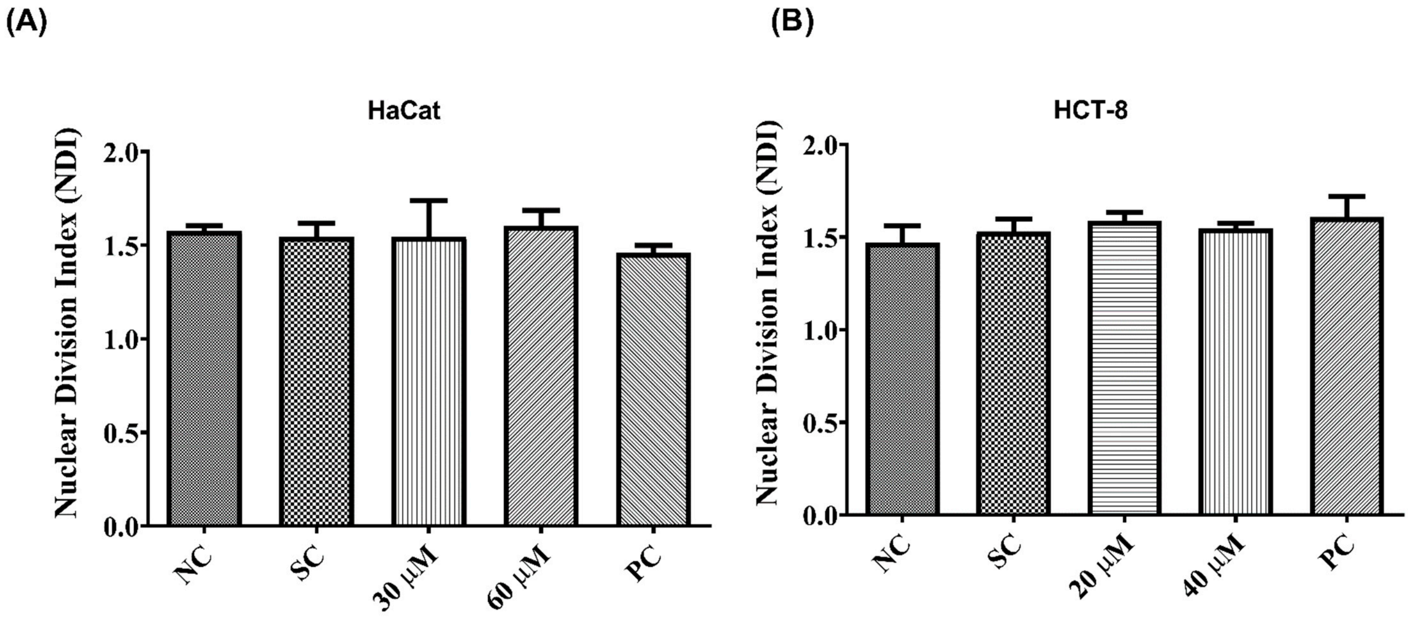

Figure S1. Nuclear Division Index determined after 48 hours of treatment with PQM-162. Five hundred binucleated cells were counted per slide resulting in a total of 1500 binucleated cells in HaCaT (A) and HCT-8 (B) cytological preparations. Values are presented as the mean ± standard deviation (SD) of three independent experiments. ANOVA analysis followed by Tukey's multiple comparisons test. HCT-8 – colorectal adenocarcinoma cell line, HaCaT – normal human keratinocytes cell line, NC – negative control, SC – solvent control (DMSO – dimethyl sulfoxide 1%), PC – positive control (5-FU – 5-fluorouracil – 0.8 μM), NDI – Nuclear Division Index, PQM-162 (*E*)-3-(4-hydroxy-3-methoxyphenyl)-*N*'-((*E*)-4-methoxybenzylidene) acrylohydrazide.
